# Impact of sub and supra-threshold adaptation currents in networks of spiking neurons

**DOI:** 10.1007/s10827-015-0575-3

**Published:** 2015-09-24

**Authors:** David Colliaux, Pierre Yger, Kunihiko Kaneko

**Affiliations:** Institut des Systèmes Intelligents et de Robotique (ISIR), CNRS UMR 7222, UPMC University Paris, 4 Place Jussieu, 75005 Paris, France; Institut d’Etudes de la Cognition, ENS, Paris, France; Sorbonne Université, UPMC University Paris06 UMRS968, Insititut de la Vision, Paris, France; INSERM, U968, Paris, France; CNRS, UMR7210, Paris, France; Department of Basic Science, The University of Tokyo, 3-8-1, Komaba, Meguro-ku, Tokyo 153-8902 Japan

**Keywords:** Adaptation, Neuronal dynamics

## Abstract

Neuronal adaptation is the intrinsic capacity of the brain to change, by various mechanisms, its dynamical responses as a function of the context. Such a phenomena, widely observed *in vivo* and *in vitro*, is known to be crucial in homeostatic regulation of the activity and gain control. The effects of adaptation have already been studied at the single-cell level, resulting from either voltage or calcium gated channels both activated by the spiking activity and modulating the dynamical responses of the neurons. In this study, by disentangling those effects into a linear (sub-threshold) and a non-linear (supra-threshold) part, we focus on the the functional role of those two distinct components of adaptation onto the neuronal activity at various scales, starting from single-cell responses up to recurrent networks dynamics, and under stationary or non-stationary stimulations. The effects of slow currents on collective dynamics, like modulation of population oscillation and reliability of spike patterns, is quantified for various types of adaptation in sparse recurrent networks.

## Introduction

Most neurons in primary sensory areas tend to change the strength of their dynamical responses over time for sustained and constant inputs, in a so-called adaptation process. The detailed mechanisms of this adaptation are still not clearly understood, and can result from various phenomena that might be combined: homeostasis or intracellular mechanisms (Turrigiano and Nelson 2004; Benda and Herz 2003), short term plasticity (Tsodyks et al. 2000), or even network-wide effect originating from lateral connections (Haider et al. 2010). In this paper, we propose to investigate computationally some effects of neuronal adaptation, from both a single-cell and a network point of view. By using a phenomenological model for neurons based on an integrate-and-fire model with intrinsic adaptation and its macroscopic counterparts, we studied the effects of slow adaptation currents on neuronal dynamics at the network level.

Detailed models of intrinsic plasticity as a source of homeostasis and neuronal adaptation have already been investigated. Such biological models are used to explore the biomechanistic effects of slow ionic channels on the microscopic cortical activity of single cells (Benda and Herz 2003; Gigante et al. 2007). On a more phenomenological level, they can be used to study the firing rates dynamics (Treves 1993) or constrain their distribution (Benucci et al. 2013) in macroscopic models of cortical networks. All those models, however, are mostly based on two types of current involved in slow adaptation and related to the flow of potassium and calcium ions through the membrane. The interaction of the adaptation currents with the neuronal dynamics is complex, impacting both the firing threshold and the behaviour of the cell (Benda et al. 2010). On one hand, voltage-gated potassium currents, like Kv1, are activated by a mild depolarization of the membrane potential and control the propagation of spikes by modulating the spike threshold (Higgs and Spain 2011), a phenomena also referred to as accommodation. On the other hand, calcium-gated potassium currents are activated only at high levels of depolarization and result in after-hyper-polarization (AHP), a drop of the membrane potential and long lasting decrease of excitability after a spike is emitted (Andrade 2011). The time scale of these adaptation mechanisms can expand over a wide range up to minutes (Pozzorini et al. 2013) and are very heterogeneously distributed among cells but especially pronounced in pyramidal cells (Nowak et al. 2003).

Combined all together, those two distinct adaptation mechanisms can prevent the saturation in the spike generation process of a neuron or modulate its synchronization properties, both acting in a different manner as shown in previous experimental (Deemyad et al. 2012) or theoretical works (Ermentrout 1998; Ermentrout et al. 2001; Prescott and Sejnowski 2008; La Camera et al. 2004). The voltage-gated sub threshold current shifts the input threshold for triggering spike depending on the basal activity: it can thus be seen as a good mechanism to explain that some response properties of a neuron are independent of the background inputs. It has also been shown to play a role in the homeostasis after sensory deprivation (Nataraj et al. 2010). Conversely, the calcium-gated current triggered by a spike does not only affect the threshold but also increase the dynamic range of the neuronal response and thus avoids saturation. Based on those observations, we will refer to those two different mechanisms of adaptation as a linear or sub-threshold one (voltage-gated channels) versus a non-linear or supra-threshold one (calcium-gated channels). The role of adaptation in enhancing reliability of spike-timing of neurons stimulated with periodic inputs was also studied in Schreiber et al. (2004). Adaptation could also act functionally as a decorrelation machine (Wang 1998).

The dynamics of cortical networks are considerably enriched with adaptation currents, inducing bursting activity, slow oscillations, and homeostasis (Tabak et al. 2000; Giugliano et al. 2004; Gigante et al. 2007). Rhythmic activity of central pattern generators involved in locomotor behaviour (Grillner 1997), or multi-stable dynamics of cortical networks when ambiguous stimulus is presented (Wilson 2003) were related to slow potassium currents. More recently it has been shown that such currents should be included to explain the rich repertoire of ongoing activity observed *in vivo* (Mattia and Sanchez-Vives 2012) and which could be the microscopic substrate of the resting state activity recorded in fMRI (Gusnard and Raichle 2001). It is therefore crucial to have a better understanding of the effect of this adaptation at a single-cell or at a population level.

In this study, we investigated those physiological observations on adaptation in a model of cortical dynamics simple enough, so that large scale simulations can be performed. To do so, we used an adaptive exponential integrate-and-fire neuron model suited for large-scale simulations of cortical networks (Brette and Gerstner 2005) and known to be complex enough to reproduce all the dynamical repertoire recorded *in vitro* in various cell types (Izhikevich 2001; Brette and Gerstner 2005). Indeed, the dynamics of networks of such units have been recently investigated in Ladenbauer et al. (2014) and Farkhooi et al. (2011) and this model have been successful in capturing more diverse dynamics by generating a slow inhibitory feedback, reflecting the fact that neurons tends to adapt when stimulated with a constant inputs. While a classical model would provide a sustained response, models with adaptation will have response closer to what is observed in biological recordings. By studying the sub-threshold (linear) and the supra-threshold (non-linear) effects of the adaptation on single-cell response or in a neuronal network, we were able to disentangle the functional role of those two components on aspects of the neuronal dynamics, like oscillations or the reliability of spike patterns.

## Materials and methods

### **Neuron model**

Simulations of the spiking neurons were performed using a custom version of the NEST simulator (Gewaltig and Diesmann 2007) and the PyNN interface (Davison et al. 2008), with a fixed time step of 0.1ms. In all simulations, we use a planar integrate and fire neuron with exponential non-linearity as introduced in Brette and Gerstner (2005). The dynamics of the membrane potential is composed of a capacitive current $C_{\mathrm {m}} \frac {dV_{\mathrm {m}}(t)}{dt}$ and a leak current −*g*_L_(*V*_m_(*t*)−*E*_*L*_), with leak conductance *g*_*L*_ and leak reversal potential *E*_*L*_. The ratio *τ*_m_ = *C*_m_/*g*_L_ gives the relaxation time constant for the membrane voltage. Spikes are generated quasi-instantaneously by active conductances rendered by an exponential non-linearity for the current $\psi (V_{\mathrm {m}}(t))={\Delta }_{T} e^{\frac {V_{\mathrm {m}}(t)-V_{T}}{{\Delta }_{T}}}$. Thus, a spike is initiated when the membrane potential *V*_m_ goes over *V*_T_ (with *V*_T_ > *E*_*L*_). The spike is cut when the voltage reaches *V*_spike_, the membrane potential is then reset to *V*_reset_.

An additional slow variable *u*, with timescale *τ*_u_, accounts for the effects of adaptation currents resulting from channels with slow dynamics. The coupling parameter *a* between *V*_m_ and *u* is a linear approximation of hyper-polarizing (*a*>0) ionic conductances such as those associated with voltage gated potassium channels. Finally, *u* is increased by an amount *b* after each spike, which models the effects of highly non-linear conductances such as those associated with calcium gated potassium channels. This results in the following system for (*V*_m_, *u*):

1 $$\begin{array}{@{}rcl@{}} C_{\mathrm{m}} \frac{dV_{\mathrm{m}}(t)}{dt}&=&-g_{\mathrm{L}} (V_{\mathrm{m}}(t)-E_{\mathrm{L}}) \end{array} $$

2 $$\begin{array}{@{}rcl@{}} &&+g_{\mathrm{L}}\psi(V_{\mathrm{m}}(t)) +I_{\text{syn}}-u \\ \tau_{\mathrm{u}} \frac{du}{dt}&=&a(V_{\mathrm{m}}(t)-E_{\mathrm{L}})-u \end{array} $$

with the spike condition:

$$\begin{array}{@{}rcl@{}} V_{\mathrm{m}}(t)>V_{\text{spike}}&\rightarrow& V_{\mathrm{m}}(t^{+})=V_{\text{reset}}\\ && u(t^{+})=u(t)+b \end{array} $$

The details of all numerical values for cell properties can be found in Table 1. These values were chosen according to those found in the literature for cortical neurons (Pospischil et al. 2011; Rossant et al. 2011; Hertäg et al. 2014). Parameters are identical for excitatory and inhibitory neurons except when specified, for example adaptation parameters are set to 0 for inhibitory cells. For every simulation, initial values of *V*_m_ are drawn from a distribution uniform in [*V*_rest_, *V*_T_].

**Table 1:** Parameters of the adaptive exponential and fire neuron used in all the simulations

Name	Value	Units	Description
*C*_m_	0.28	nF	Membrane capacitance
*g*_L_	30	nS	Total leak conductance
*τ*_refrac_	5	ms	Duration of refractory period
*V*_*T*_	−50	mV	Spike initiation threshold
*E*_L_	−60	mV	Resting membrane potential
Δ_T_	2	mV	Slope factor
*τ*_m_	9.33	ms	Membrane time constant
*V*_reset_	−60	mV	Reset potential
a	100 (E) 0 (I)	nS	adaptation conductance
b	1 (E) 0 (I)	nA	Spike-triggered adaptation current
*τ*_u_	144	ms	Adaptation time constant
*E*_rev_	(E) 0 (I) −80	mV	Synaptic reversal potential
*V*_spike_	−40	mV	Spike threshold

For parameters specific to a neuron type, E denotes excitatory cells and I denotes inhibitory cells. Note that adaptation parameters *a* and *b* are varied widely across simulations

### **Synapses**

Changes in synaptic conductances triggered by incoming spikes from excitatory and inhibitory neurons are modeled such that the total synaptic current to a neuron can be written as

3 $$ I_{\text{syn}}(t)=\sum\limits_{s \in \{exc, inh\}} (V(t)-E_{\text{rev}}^{\mathrm{s}})\sum\limits_{k} g_{s}(t-{t_{k}^{s}}) $$

**Fig. 1:**
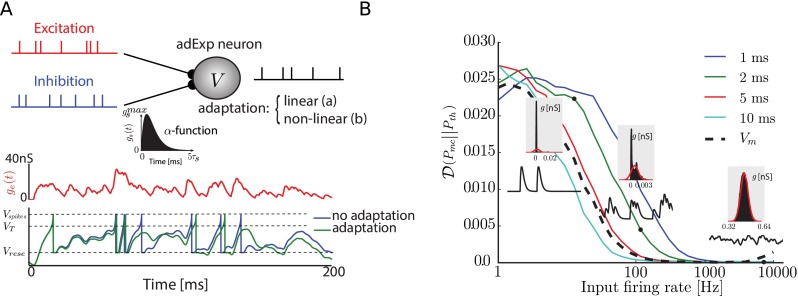
Illustrationof synaptic current and diffusion approximation. **a.** Schematic view of a neuron with the various components included in the model. Excitatory and Inhibitory inputs are modelled as *α*-shaped conductances (inset), and examples of the *V*_m_ dynamics can be seen in different cases: without adaptation or with non-linear adaptation. **b.Plain line**: KL-divergence between input simulated conductance distribution and theoretical estimate $\mathcal {N}(\mu , \sigma ^{2})$ in the diffusion limit (*ν*_exc_> > *τ*_exc_) for various synaptic time constants, assuming independent Poisson inputs at excitatory synapses. *Dashed line* KL-divergence between the recorded membrane potential distribution and its closest Gaussian distribution with same mean and variance. Insets are example of simulated conductance traces and recorded *V*_m_ distribution (in mV), compared to theoretical predictions

The times ${t_{k}^{s}}$ (*s*∈{*exc*, *inh*}) are the times of the incoming spikes, respectively at excitatory and inhibitory synapses. The dynamics *g*_*s*∈{*exc*, *inh*}_(*t*) after a spike is described by an alpha-function, from Rall synapse model (Bard Ermentrout and Terman 2010), so that the synaptic inputs may be rewritten as the convolution of the spike trains with kernels *K*^*s*^ with $K^{s}(t)=[t]_{+} e^{-\frac {t}{\tau _{s}}}/\tau _{s}$, [.]_+_ representing the Heaviside function. If the maximal conductance for a synapse type *s* is written ${g_{s}^{m}}$, we have $g_{s}(t)={\sum }_{k}{g_{s}^{m}} K^{s}(t-{t_{k}^{s}})$, with *k* running over incoming spikes and *s*∈{*exc*, *inh*}. Figure 1 is illustrating all those concepts, in a condensed form. In all the paper, we took *τ*_exc_ = 2ms and *τ*_inh_ = 3ms for the synaptic time constants.

### **Diffusion approximation**

The spiking activity for a neuron receiving only excitatory inputs at a rate *ν*_exc_ is controlled by the total effective conductance input $\mu =g^{m}_{exc}\nu _{\text {exc}}\tau _{\text {exc}}$. The minimum effective conductance input for spike to be triggered, related to the rheobase current, is the one that brings the membrane potential just above its threshold value and it is defined in the following by $g^{\text {rheo}}=\frac {g_{L}(V_{T}-E_{L})}{V_{T}}$. The deviation of the membrane potential from the diffusion approximation is measured by the Kullback-Leibler divergence between the simulated membrane potential *P*_*mc*_ and a theoretical Gaussian distribution *P*_*th*_ having mean and variance as predicted from the diffusion approximation:

$$\mathcal{D}(P_{mc}||P_{th})=\int\limits_{-\infty}^{\infty} P_{mc}(v) ln \frac{P_{mc}(v)}{P_{th}(v)} dv. $$

### **Adaptation**

In all the manuscript, we consider the sub-threshold or linear part of the adaptation as the one controlled by the *a* parameter in the equation of the adaptive exponential neuron, and the supra-threshold or non-linear part of the adaptation as the one controlled by the *b* parameter. Therefore, a neuron with only linear adaptation is one with *b* = 0, and one with only non-linear adaptation has *a* = 0. Physiological interpretation of these parameters is discussed in the neuron model description.

### **Cortical column**

A column is composed of two populations of excitatory and inhibitory neurons connected in a random manner (Erdös-Renyi wiring) with excitatory and inhibitory weights *g*_exc_ and *g*_inh_, and receiving external input *ν*_ext_ from spike source generated through Poisson processes with weights *g*_ext_. This is equivalent to a so-called sparse balanced network (Brunel 2000). Neuron parameters are the same as those described in Table 1. A schematic drawing of the column can be found on Fig. 4a. More precisely, the sizes of the populations are *N*_exc_ = 800, *N*_inh_ = 200 and *N*_ext_ = 200 (a classical 4:1 ratio between excitatory and inhibitory cells). Connections among neurons are drawn randomly with probabilities *p*_*AB*_, *A* (and *B*) being populations from which input (and output) neurons are selected (*E* for excitatory, *I* for inhibitory, *ext* for external input). In and out degrees of neurons are thus distributed according to a Poisson law with parameter *p*_*AB*_*N*_*A*_*N*_*B*_. In all the simulations, we use a value of *p*_*EE*_ = *p*_*EI*_ = 0.05, *p*_*II*_ = *p*_*IE*_ = 0.05 and *p*_*ext*_ = 0.01. Excitatory weights are fixed to *g*_exc_ = 6nS and inhibitory weights are modified through the control parameter *g* defined by *g*_inh_ = *g*.*g*_exc_. For external connections, we have *g*_ext_ = *g*_exc_/6. Delays of the connection are all equal to the simulation time step, i.e. 0.1ms.

### **Classification of dynamical regimes**

The column is considered to be in a Synchronous regime if the pairwise spike correlations 〈*CC*(0)〉 are over a 0.026 threshold value. Pairwise spike correlations are computed as the mean Pearson correlation coefficient averaged over *N* = 10000 pairs of randomly chosen cells. The area for saturated regime with Synchronous regular dynamics correspond to an average firing rate over 75 Hz and a mean coefficient of variation for the interspike intervals (CV ISI) less than 0.2. Silent regimes correspond to firing rates lower than 0.2 Hz. Regions of the diagram not detected by these criteria are denoted as the Asynchronous Irregular regime.

### **Reliability of responses**

The reliability of the response is assessed by considering repeated input spike trains from a population of 2000 neurons connected with probability *p*_*ext*_ = 0.01 to the excitatory cells of the column. For inputs as homogeneous Poisson process, we consider spike trains with firing rate, *r*_*cst*_ = 85*Hz* and for inputs as inhomogeneous Poisson processes, the firing rate is modulated by a sine function, *r*_*var*_ = *f*_0_+*f*_1_*cos*(2*πωt*) with *f*_0_ = 100*Hz*, *f*_1_ = 40*Hz* and *ω* = 5*Hz*. The spiking response and the average membrane voltage is recorded for 40 times and measures are averaged on these 40 repetitions of the same input spike trains.

The reliability of the spiking response is monitored by the pairwise spike correlation averaged over pairs of repetitions. To assess the reliability of membrane potentials, we monitor the Coefficient of Variation (CV) of the mean voltage *V*_*c*_ of excitatory neurons, that is the standard deviation divided by the mean over the 40 repeated trials, and is unitless. The spike and voltage reliability measures are averaged over 10 different inputs and reported with standard deviations for various values of *a* and *b* in Fig. 9.

### **Mean field**

The coarse-grained dynamics of a column can be captured by the firing rates (*r*_*E*_, *r*_*I*_) averaged over excitatory and inhibitory populations. The evolution equation for the firing rate dynamics is then reduced to the Wilson-Cowan system:

4 $$ \left\{\begin{array}{l} \tau_{E} \frac{dr_{E}}{dt} = -r_{E}+f_{E}(w_{EE} r_{E} \tau_{E} -w_{IE} r_{I} \tau_{I})\\ \tau_{I} \frac{dr_{I}}{dt} = -r_{I}+f_{I}(w_{EI} r_{E} \tau_{E} -w_{II} r_{I} \tau_{I}) \end{array} \right. $$

with *w*_*AB*_ standing for the effective coupling from the sub-population *A* to the sub-population *B* ($w_{AB}=p_{AB}N_{A} N_{B} {g_{A}^{m}}$, *p*_*AB*_ probability of connection from *A* to *B*, *N*_*A*_ number of neurons in population *A*, ${g_{A}^{m}}$ conductance level at a synaptic contact), *τ*_*A*_ the synaptic time constant of the sub-population *A* mentionned in the neuron model and *f*_*A*_ the firing rate response of sub-population *A*. The function *f*_*A*_(*I*) is the response curve, that is the mean firing rate of neurons in sub-population *A* when stimulated by incoming inputs *I*. These response curves and their approximations are discussed in the results Section 3.1. We are using standard numerical methods of bifurcation theory as implemented in the AUTO library with the python interface PyDSTools (Clewley 2012). The local stability analysis for the fixed points of the dynamical system consists in the study of the set of parameters where some eigenvalues of the Jacobian are zero. Further study of the Jacobian are indicative of the type of instability at these points (Kuznetsov 1998). A fold (or saddle-node) bifurcation curve separates regions with one stable fixed point from bistable regions with two stable fixed points separated by an unstable fixed point. Along those curves, the Jacobian has a zero eigenvalue and a stable fixed point and an unstable fixed point annihilate. When an eigenvalue is non-zero but has a zero real part, there is a Hopf bifurcation curve separating a region with one stable fixed point from a region with a limit cycle. On those curves, eigenvalues of the Jacobian are a pair of pure conjugated numbers. If 2 saddle-node branches collide in the parameter plane, the resulting bifurcation point is a so-called cusp. Other bifurcations points are related to Hopf bifurcations when the linear field cancels (Bogdanov-Takens point) or when the quadratic contribution to the vector field cancels (Bautin point). Note that Bautin points separate the part of the Hopf curve where the limit cycle arising has infinitesimal amplitude from the part where it has finite amplitude. Simulations of the stochastic dynamics where implemented in C++ using the Milstein scheme.

## Results

### Dynamics of a single neuron without adaptation

First, we consider the case of a single adaptive exponential integrate-and-fire neuron neuron simply bombarded with excitatory and inhibitory homogeneous Poisson inputs. Both types of inputs are triggering conductances changes at the soma level as illustrated on Fig. 1a, and the spiking activity of the neuron is modified by the adaptation mechanisms regulating the activity. In all the following, we assume that “linear” (or sub-threshold) adaptation is mediated by the parameter *a* of the model (see Section 2), while “non-linear” (or supra-threshold) adaptation is related to the *b* parameter. To gain a better understanding of the responses, we consider the spiking activity of the neuron without any adaptation (*a* = 0, *b* = 0, see Section 2). Without a loss of generality, we can restrict the analysis to the case where only excitatory inputs are impinging the cell (*ν*_inh_ = 0). In this case, the average synaptic input onto the cell for filtered Poisson spike train of rate *ν*_exc_ with *ν*_exc_*τ*_exc_>>1 can simply be approximated by a Gaussian process of distribution *G*_exc_ = *N*(*μ*, *σ*^2^) with a mean *μ* and a variance *σ*^2^ (Papoulis 1965; Ricciardi and Sacerdote 1979; Lánský and Lánská 1987; Richardson and Gerstner 2005; Hertäg et al. 2014) (see Section 2 for the synapse model):

5 $$ \left\{\begin{array}{rcr} \mu &=& \nu_{\text{exc}} {\int}_{0}^{\infty} K^{\text{exc}}(\tau)d\tau = g^{m}_{\text{exc}} \tau_{\text{exc}} \nu_{\text{exc}}\\ \sigma^{2} &=& \nu_{\text{exc}} {\int}_{0}^{\infty} (K^{\text{exc}}(\tau))^{2}d\tau = (g^{m}_{\text{exc}})^{2} \tau_{\text{exc}} \nu_{\text{exc}} \end{array}\right. $$

The validity of such a diffusion approximation can be checked by computing the Kullback-Leibler divergence (KL, see Section 2) between the theoretical distribution of the synaptic input conductances (*G*_exc_) and numerical simulations for various values of the maximal conductance $g^{m}_{\text {exc}}$ and input firing rate *ν*_exc_, keeping the total mean effective input $\mu =g^{m}_{\text {exc}} \tau _{\text {exc}} \nu _{\text {exc}}$ constant. In all the numerical simulations, we use the planar adaptive integrate and fire neuron described in Brette and Gerstner (2005) and further studied in Touboul and Brette (2008).

As we can see from Fig. 1b, in absence of adaptation and for our particular model, this diffusion approximation on *G*_exc_ becomes valid for a synaptic time constant of *τ*_exc_ = 2 *ms*., and for input rates of approximately 1000Hz (KL ≃ 0). This can be seen in the insets by the convergence of the conductance distribution toward a Gaussian distribution, while incoming synaptic conductances are shown as black traces. Similarly, the KL-divergence between the empirically measured distribution of the *V*_m_ and a Gaussian distribution having mean *μ*_*V*_ and variance ${\sigma _{V}^{2}}$ of the real *V*_m_ distribution (dashed line in Fig. 1b) is converging toward zero. The voltage being filtered with a slower timescale (in the order of *τ*_m_≈10 *ms*, see Section 2), converges faster than for the conductance distribution. Therefore, this diffusion approximation is valid for a neuron receiving input spikes at realistic rate (1–10Hz) from a population as small a 1000 neurons. A reasonable size of a population from which a mean field model can be used is needed to describe its dynamics and to justify the size of the cortical columns we will use in the following.

Then, it is known that for a stationary Poisson input at a firing rate *ν*_exc_ in the diffusion limit, i.e for low values of $g^{m}_{\text {exc}}$ and approximated by a Gaussian process $\mathcal {N}(\mu , \sigma ^{2})$, individual fluctuations in *V*_m_ are small compared to *V*_*T*_. Therefore, the synaptic current $I_{\text {exc}}(t)=G_{\text {exc}}(t)(V_{\mathrm {m}}(t)-E^{exc}_{\text {rev}})$ can also be approximated by a Gaussian process with mean $\hat {\mu } = \mu (V_{T}-E^{exc}_{\text {rev}})$ and variance $\hat {\sigma }^{2}=\sigma ^{2} (V_{T}-E^{exc}_{\text {rev}})^{2}$ (Destexhe et al. 2001; Richardson and Gerstner 2005). The dynamics for the exponential integrate-and-fire neuron can be summarized by the adaptive integrate-and-fire Ornstein-Uhlenbeck process defined as follows

6 $$\begin{array}{@{}rcl@{}} \tau_{\mathrm{m}} \frac{dV_{\mathrm{m}}(t)}{dt} &=& -(V_{\mathrm{m}}(t)-E_{\mathrm{L}})+ \psi(V_{\mathrm{m}}(t)) \end{array} $$

7 $$\begin{array}{@{}rcl@{}} && +\frac{\hat{\mu}+\hat{\sigma}\sqrt{\tau_{m}}\eta(t)}{g_{\mathrm{L}}} \end{array} $$

where *η*(*t*) is drawn from a Gaussian distribution $\mathcal {N}(0, 1)$.

**Fig. 2:**
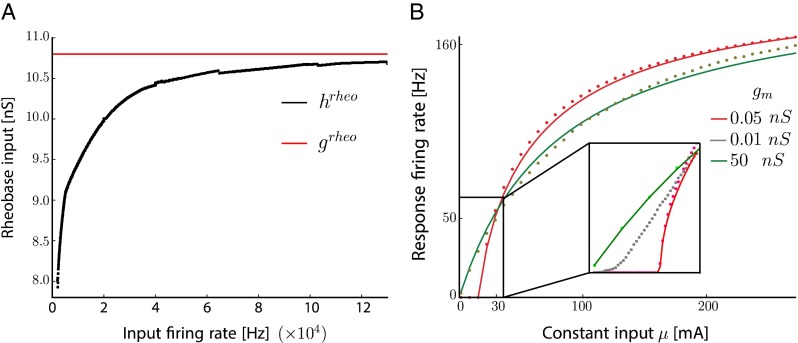
**a.** Difference between the real rheobase conductance *g* ^rheo^ and its estimated value *h* ^rheo^ from diffusion approximation as a function of the rate of excitatory Poissonian input. **b.** Firing rate of the neuron without adaptation subject to excitatory inputs from a Poisson source as a function of the input *μ*, for several maximal conductances $g^{max}_{exc}$. *Red curve* is for low membrane conductances values and high firing rates, while *green curve* is obtained for high conductances and low firing rates. *Plain lines* are theoretical approximations obtained for the situations (low rate, high conductance) (*green*) and (high rate, low conductance) (*red*), dots are obtained from numerical simulations. Inset shows simulations of couples (rate, conductance) for several values of *g*_*m*_., color-coded

For this model, as already studied in La Camera et al. (2004) the rheobase effective input *g*^rheo^, i.e. the minimum conductance input triggering a spike, is a fixed value that can be compared with estimations obtained from numerical simulations. As we can see on Fig. 2a, for low input firing rate *ν*_exc_, the neuron tends to fire spikes at lower values *h*^rheo^ of input threshold than the theoretical one but as the input firing rate is increased, the fluctuations in synaptic inputs vanish and the neuron tends to behave as its deterministic limit *g*^rheo^. From Fig. 2a, we conclude that this deterministic limit, reached at around 5000*Hz*, is more restrictive than the one given by the diffusion approximation alone, but remains valid for the networks considered in the following.

The two limiting cases of large and small conductance *g*_*exc*_ can be studied while maintaining the mean effective input *μ* = *g*_*exc*_*ν*_*exc*_*τ*_*exc*_ constant. When the maximal conductance is close to $g^{max}_{\text {exc}}$, response is linear at low inputs rates and saturates to the maximal firing rate imposed by the refractory period, with $\nu _{\text {out}}=\frac {\nu _{\text {exc}}}{1 + \tau _{\text {ref}} \nu _{\text {exc}}}$ (see green curve on Fig. 2b). On the opposite, in the limit of high firing rates, *ν*_exc_≫1/*τ*_exc_, the sub-threshold dynamics is well approximated by the Ornstein-Uhlenbeck process shown above, and it is known Ricciardi and Sacerdote (1979) and Sacerdote and Giraudo (2013) that the mean first passage time is given by

$$T_{OU} = \sqrt{\frac{\pi \tau_{\mathrm{m}}}{\hat{\sigma}^{2}}} {\int}_{V_{\text{reset}}-\hat{\mu} \tau_{\mathrm{m}}}^{V_{\text{spike}}-\hat{\mu} \tau_{\mathrm{m}}} \{1+Erf(z/\hat{\sigma} \sqrt{\tau_{\mathrm{m}}})\} e^{\frac{z^{2}}{\hat{\sigma}^{2}}}dz $$

where *Erf* is the error function and other parameters are described in the Methods (see Richardson and Gerstner 2005, Ostojic and Brunel 2011 for a fast method to compute this quantity). The firing rate for the adaptive exponential neuron without adaptation is then simply *ν*_out_ = 1/(*τ*_*ref*_+*T*_*OU*_) (see red curve on Fig. 2b). Note that in the inset of Fig. 2b firing rate response are plotted as a function of *g*_*exc*_ ranging from 0.05 to 50 nS, color-coded. Response behaves linearly for $g_{exc} \simeq g^{max}_{\text {exc}}$. Interestingly, for values of *g*_*exc*_ in between, response curves cross almost all in the same region around which the response firing rate is not dependent on the size of post-synaptic potential for a given effective input. This could be interesting for networks including heterogeneities of synaptic weights as their output rate would be similar for identical effective input when input rates are scaled appropriately.

### Dynamics of a single neuron with adaptation

Now if we consider adaptation, its dynamics (*τ*_u_ = 100ms) is slower than timescales involved in spike generation or membrane relaxation (*τ*_m_ = 10ms) so for the analysis of response properties it is possible to assume timescale separation (van Vreeswijk and Hansel 2001; Benda and Herz 2003; Ermentrout 1998). Let us first study the effect of linear (sub-threshold) adaptation current on the response to Poisson input spike train. At slow time scale, the fast membrane dynamics can be averaged so that $\langle (V_{\mathrm {m}}(t)-E_{L})\rangle =\frac {\hat {\mu }}{g_{L}}$ and injecting this value in the dynamics for the adaptation current gives the stationary current $u=a \frac {\hat {\mu }}{g_{L}}$ resulting in the effective dynamics for *V*_m_(*t*)

8 $$\begin{array}{@{}rcl@{}} \tau_{m} \frac{dV_{\mathrm{m}}(t)}{dt}&=&-g_{\mathrm{L}} (V_{\mathrm{m}}(t)-E_{\mathrm{L}})+g_{L} \psi(V_{m}) \\ &&+(1-a)\frac{\hat{\mu}}{g_{L}} + \frac{\hat{\sigma}}{g_{L}} \eta(t) \end{array} $$

The effect of linear adaptation is thus to reduce the effective input and the firing rate response for all inputs, shifting the response curve. This negative feedback could be useful to tune the threshold depending on the basal level of computation, and a similar mechanism has been shown to enable contrast invariant computations in the visual system (Carandini and Ferster 1997).

If we now consider the non-linear effect of adaptation, we can notice that between two consecutive spikes *t*_*k*+1_−*t*_*k*_ = *T*, a discrete map describes the dynamics of the adaptation current. At each spike time, we have $u(t) \rightarrow u(t)e^{-T/\tau _{u}} + b$, so that between 0 and T we have $u(t)=b(1-e^{-t/\tau _{u}})/(1-e^{-T/\tau _{u}})$. For a high firing rate *ν*_*exc*_ compared to the adaptation time scale *τ*_*u*_, the average adaptation current can therefore be estimated as 〈*u*〉=*τ*_*u*_*bν*_*exc*_. Figure 2b (red curve) showed that for *ν*_*exc*_ far below saturation, the response curves of the neuron without adaptation behave linearly. In this linear regime of the response, the firing rate can therefore be described with a linear relationship $\nu _{\text {out}}=k(\hat {\mu }-\hat {\mu }_{c})$, with $k, \hat {\mu }_{c}$ being constants and $\hat {\mu }$ the total current to the membrane $\hat {\mu }=I_{syn}-u$. By replacing *u*(*t*) by its mean value estimated above, we have $\nu _{\text {out}}= k (I_{syn}-\hat {\mu }_{c})/(1+kb\tau _{u})$, such that the net effect of non-linear adaptation is to change the slope of the response to slow input variations, as also found in Ermentrout (1998) and Benda and Herz (2003).

**Fig. 3:**
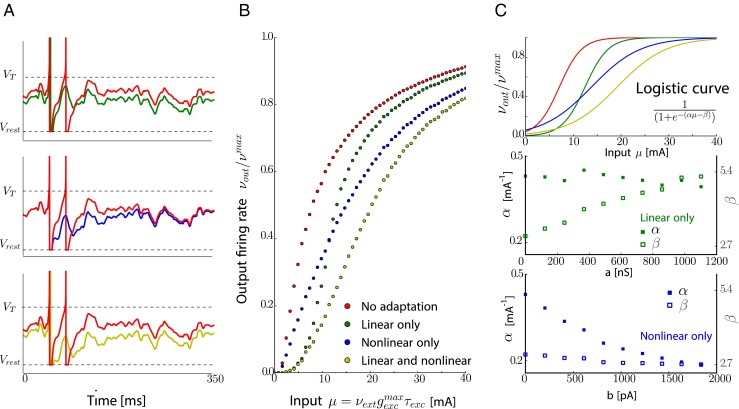
Influence of linear and non linear adaptation **a.** Comparison of the response to the same excitatory input from a Poisson spiking process for a neuron without adaptation and neuron with (*up*) linear adaptation (*middle*) non linear adaptation (*bottom*) combined linear and non linear adaptation conditions (see Section 2). **b.** Firing rate response as a function of the input rate in the 4 conditions previously listed. **c.Top**: Sigmoidal rate function used to estimate the effect of adaptation. *Middle*: Effect of linear adaptation on threshold (*β*, unfilled square markers) and gain (*α*, filled square markers) of the sigmoid. *Bottom*: same as middle, but when the non-linear adaptation is varied

Figure 3a shows examples of the dynamics of the exact same neuron, in different conditions of adaptation. We compare single-cell responses to a particular input without any adaptation (*a* = 0, *b* = 0) to a regime with full adaptation (*a*, *b* with standard values as defined in Section 2), one with only linear adaptation (*b* = 0), and one with only non-linear spike frequency adaptation (*a* = 0). In Fig. 3b, the firing rate responses as functions of the input *μ* are plotted for those different adaptation conditions. The effect of linear adaptation is a shift in the spike threshold (see Fig. 3b, the fact that when *b* = 0 (green curve), the curve is rising for higher values of *μ* compared to the response curve with no adaptation (in red)). Similarly, the effect of non-linear adaptation (*a* = 0) is equivalent to a change in the slope of the response curve to slow input variations (see blue curve). Schematically, we can fit with a least square fitting procedure those response curves to sigmoidal functions $f(\nu _{\text {ext}})=1/(1+e^{-\alpha (\nu _{\text {ext}} -\beta )})$ (see Fig. 3c, top) and quantify this behavior.

Using the sigmoidal fits, we study the variation of the thresholds *β* and the gains *α*, as functions of the adaptation parameters of the neuron, *a* and *b*. We can see on Fig. 3c, middle, that the linear adaptation (controlled by *a*) affects mostly the threshold *β* shifting the response curve to the right with higher values of *a* resulting in higher thresholds *β*. This dependence is linear as expected from the analysis via time scales separation. On the other hand, the non-linear adaptation (controlled by the parameter *b*) affects mostly the gain *α* of the sigmoidal response curve by decreasing the slope of the response function, with higher values of *b* resulting in lower *α* (see Fig. 3c, bottom). Again, as expected from former considerations, this dependence is non-linear. The decrease in gain also results in a wider dynamic range. Note that the change in *α* is necessary to keep the rheobase constant so that non-linear adaptation changes both *α* and *β*. We note that having lower gain, the input range for which linear approximation of the response is valid is increased and this can be summarized by stating that the net effect of non-linear adaptation is to linearize the response curve.

### Dynamics of a cortical column with adaptation

**Fig. 4:**
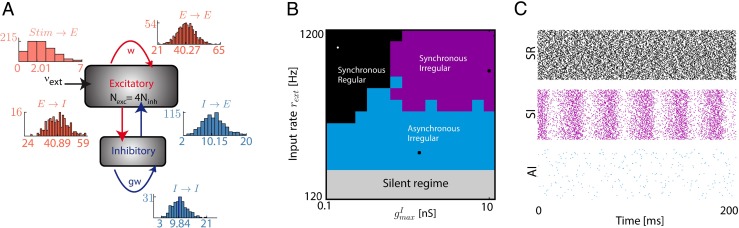
Structure and dynamics of the cortical column model **a.** Schematics of the wiring connections in the cortical column. Two populations of 800 excitatory (*red*) and 200 inhibitory (*blue*) neurons are reciprocally connected with a probability of 5 %. Excitatory cells are also stimulated by external inputs at a rate *ν*_ext_. Insets are showing the distribution of the indegree for all the different connections between populations involved in the column. **b.** Phase diagram as a function of external firing rate *ν*_ext_ and inhibitory maximal conductance (when *g* is varied), without any adaptation. We can see four distinct regimes of activity. **c.** Typical spike rasters for the three non-silent regions of the phase diagram

In the neocortex, neurons are arranged in complex microcircuits affecting their response properties and giving rise to internal dynamics. The cortical column (Horton and Adams 2005) is a good example of such canonical circuits encountered in the brain and has been shown to give rise to oscillatory rhythms and self-sustained irregular activity (Brunel and Hakim 1999; Vogels et al. 2005). If the fine connectivity details of such a column are still poorly understood (Binzegger et al. 2007), we can still understand the dynamical properties of a homogeneous balanced network of adapting neurons, arranged in a columnar fashion. To this aim, we simulated what we called a cortical column composed of two populations of 800 excitatory and 200 inhibitory neurons connected in a random manner with an averaged probability of connection of 5 % (Renart et al. 2010; Brunel 2000), and receiving external input from spike source generated through Poisson processes. Such a generic and classical network is often referred to in the literature as a random balanced network (Brunel 2000; Vreeswijk et al. 1996), and has been well used as a good model of *in vivo* activity in sensory cortices. More details could be found in the corresponding section of the Section 2. Schematically, the structure of the network is represented in Fig. 4a. Excitatory weights are fixed to *g*_exc_ = 6nS and inhibitory weights are modified through the control parameter *g* such that *g*_inh_ = *g*.*g*_exc_. Note that on Fig. 4a, insets shows the distribution of the indegree for all the different connections.

In Fig. 4b, we show the phase diagram of such a cortical column without any adaptation when the external rate *ν*_ext_ and the balance *g* between excitation and inhibition are varied. We can see that the dynamics of the cortical column can be maintained in an Asynchronous Irregular regime (AI, Section 2 for details on the classification) with low firing rate when external input is small enough, as already found (Brunel 2000). This large region is termed Asynchronous Irregular because neurons are firing irregularly (high coefficient of variation of their inter spikes intervals), and the network’s firing rate displays no clear oscillations. Similarly, we can see in the diagram a Synchronous Regular (SR) or a Synchronous Irregular (SI) regime. It is commonly assumed that the AI regime is a good candidate for describing cortical dynamics observed *in vivo* and it has been identified as the operating point of the brain with transient perturbation leading to amplified response with fast recovery to the resting operating point (Renart et al. 2010; Stimberg et al. 2009). Canonical raster plots of the three main dynamical regimes identified in the phase diagram are shown in Fig. 4c. In such AI regime, when input is increased, the dynamics of the network depends on the dominant polarity of the synaptic current. For an excitation dominated column, increasing input destabilizes the asynchronous irregular state so activity is amplified to a persistent saturated state with all neurons firing at their maximal frequency. For an inhibition dominated network, the destabilization of the irregular state through increased stimulation leads to oscillatory dynamics with neurons firing synchronously.

**Fig. 5:**
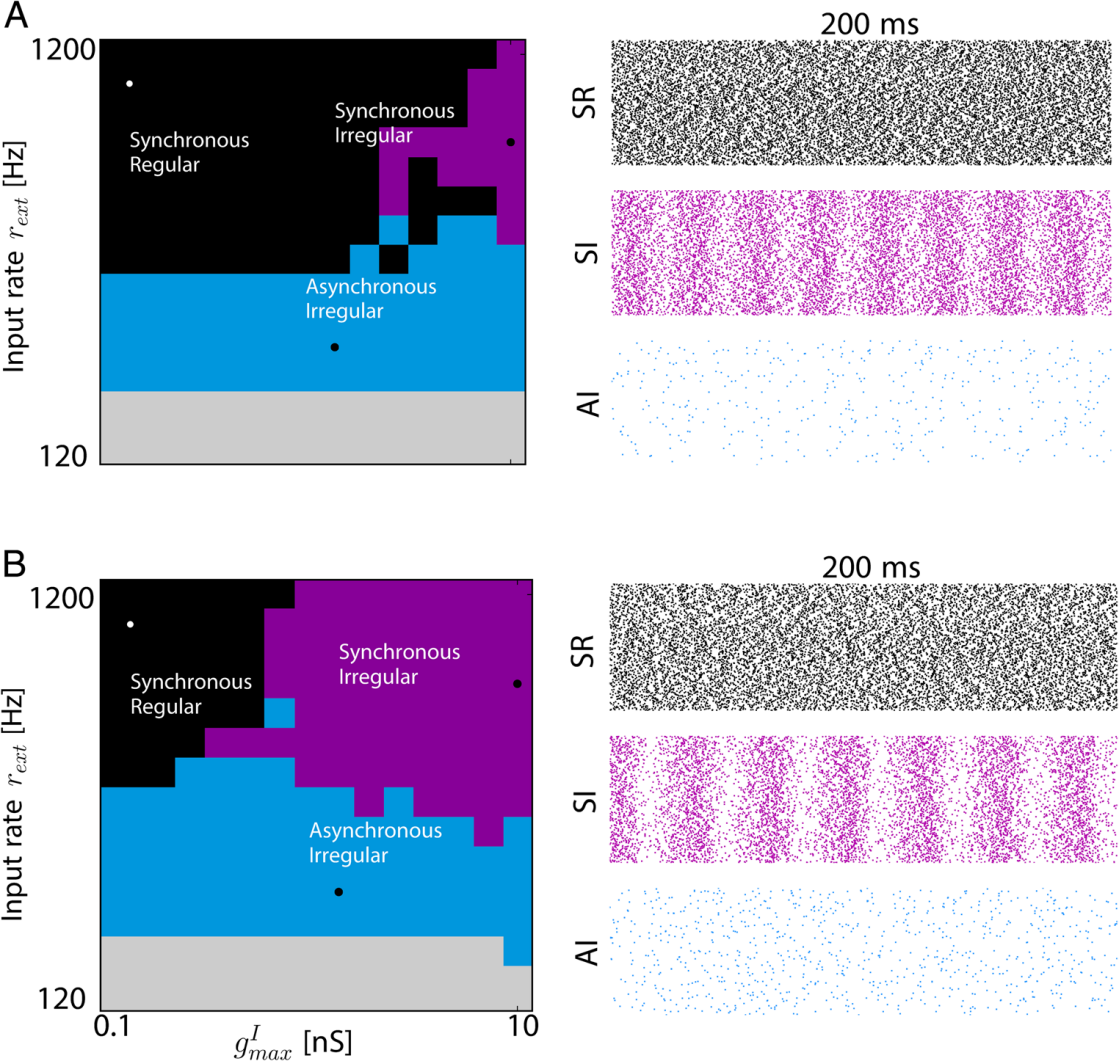
Dynamics of the cortical column model with adaptation. **a.** Phase diagram as a function *ν*_ext_ and *g*, and raster plots of the three non-silent regions with linear adaptation only **b.** Same as in **a.**, but with only non-linear adaptation

We can see what is the direct effect of the two previously discussed components of adaptation (linear v.s non-linear) in Fig. 5. As we can notice in Fig. 5a, when linear adaptation is turned on, the Synchronous Irregular region of the phase diagram tends to disappear, while the overall structure of the diagram is preserved in case of non-linear adaptation. In both cases, because the average firing rates in those regions is similar (data not shown), it is really the influence of single-cell response curves that is affecting the phase diagram. Linear adaptation, responsible for a shift in the response threshold is decreasing the synchronous activity, thus reducing the amount of correlations in the network.

### Mean field description of the cortical column

To get a better understanding on how adaptation is impacting the phase diagram, we used the fact that the coarse-grained dynamics of the cortical column can be captured, at a macroscopic level, by describing the firing rates (*r*_*E*_, *r*_*I*_) averaged over homogeneous excitatory and inhibitory neurons. Using a mean field approach, as in Tabak et al. (2000) and Giugliano et al. (2004), the evolution equation for the firing rate dynamics can be reduced to the Wilson-Cowan system:

9 $$\begin{array}{@{}rcl@{}} \tau_{E} \frac{dr_{E}}{dt}&=&-r_{E}+f_{E}(w_{EE} r_{E} \tau_{E} -w_{IE} r_{I} \tau_{I}) \\ \tau_{I} \frac{dr_{I}}{dt}&=&-r_{I}+f_{I}(w_{EI} r_{E} \tau_{E} -w_{II} r_{I} \tau_{I}) \end{array} $$

**Fig. 6:**
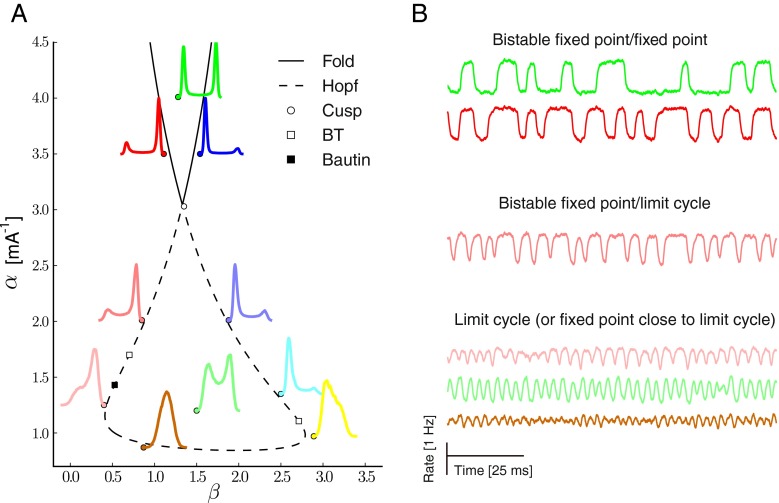
Mean Field dynamic of the cortical column with adaptation **a.** Diagram showing bifurcations of codimension 1 and 2 as a function of threshold *β* and gain *α* of the excitatory response function. The stationary distribution for the activity of the excitatory population under weak stochastic perturbation is also depicted at various points close to the instability curve. **b.** Dynamics, as a function of time, of the excitatory population at various points in the phase space

For more details, see Section 2. To describe the dynamics, we keep the sigmoidal approximation discussed in the previous section for the response curve $f_{A}(x)=(1+e^{-\alpha (\nu _{\mathrm {A}} -\beta )})^{-1}$. Beyond a fixed point attractor, this system is known to exhibit multistability and limit cycle. The transition among these regimes can be studied through a bifurcation analysis (see Section 2 for details about the related numerical tools). For this matter, as linear and non-linear adaptation were shown to affect the threshold *β* and the gain *α* of the response curves, these parameters are taken as bifurcation parameters in Fig. 6 to study the various possible instabilities.

Figure 6 depicts the distributions and the temporal traces of the activity for the excitatory sub population in the system under stochastic perturbation close to various bifurcation points (see Methods for a description of the numerical tools used for bifurcation analysis). Close to saddle-node points, the distribution is bimodal with a bias toward up or down state depending on which side of the bistable region is considered (low or high *α*). Similar bistability is observed between a limit cycle and a fixed point of high, low or moderate level of activity close to the Hopf curve. A prediction of the model is that if sufficient linear adaptation, affecting the threshold parameter *β* in the coarse-grained model, is at work in the excitatory cell dynamics, a column which would be initially oscillatory or bistable would be silenced to a low activity level in an abrupt fashion. The effect of non-linear adaptation, affecting the gain parameter *α*, is more subtle with possible transitions along the vertical axis from bistable regime in the upper cusp to various oscillatory regimes, changes in the oscillatory pattern or transition from oscillation to fixed point.

From Fig. 6 we see that the crossing of the Hopf curve is reached with a reasonable change of the threshold value *β* (related to linear adaptation) whereas a large change in gain *α* (related to nonlinear adaptation) is necessary to escape from the oscillatory region. This is consistent with previous simulations of spiking neurons where we observed that linear adaptation reduces greatly the synchronous irregular region of the diagram (see Fig. 5).

### Dynamical response to external stimuli with or without adaptation

#### Oscillatory dynamics

**Fig. 7:**
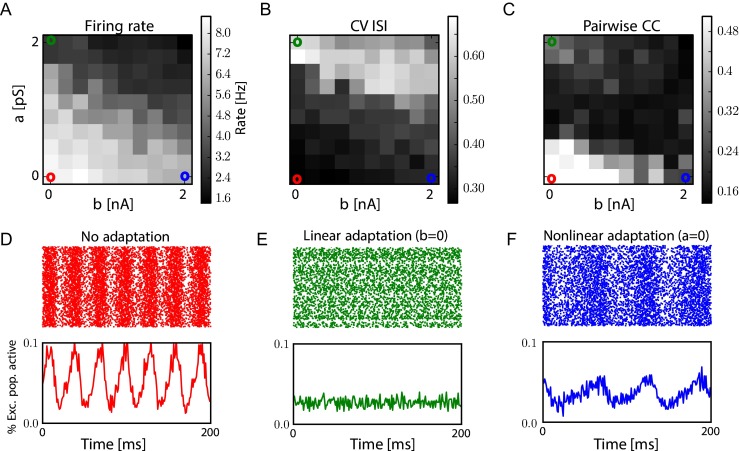
Influence of adaptation on the columnar dynamics. **a.** Measures of the network activity depending on linear and non linear adaptation parameters (*a*, *b*). Lower point in the network is set to the parameters of purple sample from Fig. 4 **d, e, f.** Samples of network activity (spike raster and mean rate) in no adaptation, linear adaptation and non linear adaptation conditions

To study the effect of adaptation on columnar dynamics modeled with spiking neurons (see Section 2), the network conductances were set so that the mean firing rate of the network is oscillatory when no adaptation is considered (*a* = 0, *b* = 0) and adaptation parameters were then varied from this setting. The measures of the activity of the excitatory sub population (firing rate, coefficient of variation of interspike) depending on those parameters are shown on the phase diagrams of Fig. 7a, b, c when the two components *a* and *b* of the adaptation are varied. As expected, when linear or non-linear adaptation is increased the mean firing rate activity is decreased but the effects of adaptation is different on the second order statistics for the dynamics at network level. The pairwise spike correlations in the network drop abruptly when linear adaptation is varied (see panel c) whereas much smoother transition is observed when non-linear adaptation is varied. Furthermore, when *b* is increased, the average firing rate in the column is oscillating at half the period of the original network. We can see on Fig. 7d, e, f examples of the activity in the three canonical regimes: without adaptation, with only linear adaptation (*b* = 0), or with only non-linear adaptation (*a* = 0).

**Fig. 8:**
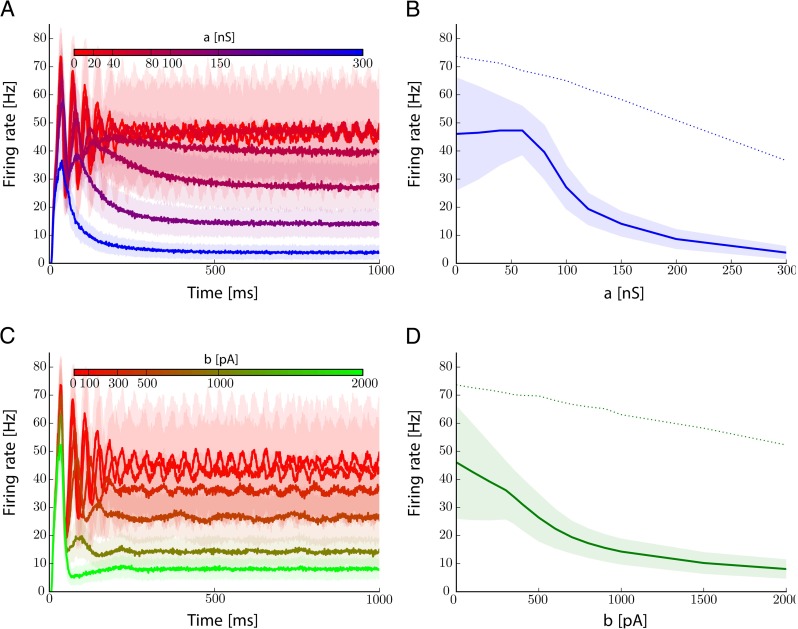
Columnar dynamics under sustained Poissonian input at constant rate. **a.** Time course of the mean firing rate of of cortical column for various levels of adaptation currents coded in colors when linear adaptation only is varied (*b* = 0). The *shaded* areas represent two standard deviation around the mean. **b.** Firing rate for various levels of adaptation currents: *solid line* is the stationary mean and shaded areas represent two standard deviations. The *dotted line* is the peak response right after the stimulus is presented. **c., d.** Same as **a., b.** but when only non-linear adaptation is varied (*a* = 0)

For a better grasp at the effects of adaptation on the dynamics of a column, we consider the time course of the activity when a stimulus is presented starting from quiescent state. The resulting responses are reported on Fig. 8. As expected from time averaged measures, after the stimulus was presented, the mean firing rate of the column oscillates when no adaptation is considered. Strong adaptation currents result in relaxation to a stationary firing rate. This relaxation occurs at the slow time scale imposed by the adaptation current for linear adaptation whereas it occurs much faster, within an oscillation cycle, for non-linear adaptation currents. To summarize, with subthreshold adaptation *a*, oscillatory fluctuations first decrease in amplitudes and then for higher values of *a* the column firing rate also decreases. In contrary, with suprathreshold adaptation *b*, both oscillatory fluctuations, oscillation frequency and mean firing rate of the columns are decreasing simultaneously.

We showed through theoretical arguments that effects of adaptation on the neuronal dynamics may be related to *α* and *β* parameters in the macroscopic model under the diffusion approximation and slow adaptation. The study of the 2 variables macroscopic model is then useful to interpret some aspects of the dynamics like oscillations. There are aspects of changes induced by adaptation which are not discussed in this way because they would go beyond the 2 variables mean field model we consider to discuss qualitative dynamics. For instance chaotic dynamics where found in a macroscopic model including short term plasticity in Cortes et al. (2013).

#### Reliability of spike patterns

In previous sections, we analyzed the stationary responses of a neuronal network with adaptation, and we distinguished the linear v.s the non-linear part of that mechanism, either with simulations of spiking neurons or with a macroscopic approximation. Now we consider the dynamical responses of the same cortical column, but when stimulated with a Poisson process at constant rate, or with one with rate modulated in a sinusoidal manner (see Methods). For both scenarios, we observed the effect of adaptation on the reproducibility of the responses, when the exact same realization of the input spike patterns were replayed to the exact same network. At each trial, initial conditions of the network were different (see Methods), and to visualize the mean response of the column, we computed and plotted the mean voltage over all *N* neurons within the column, *l*(*t*)=<*V*_*m*_(*t*)>_*N*_.

**Fig. 9:**
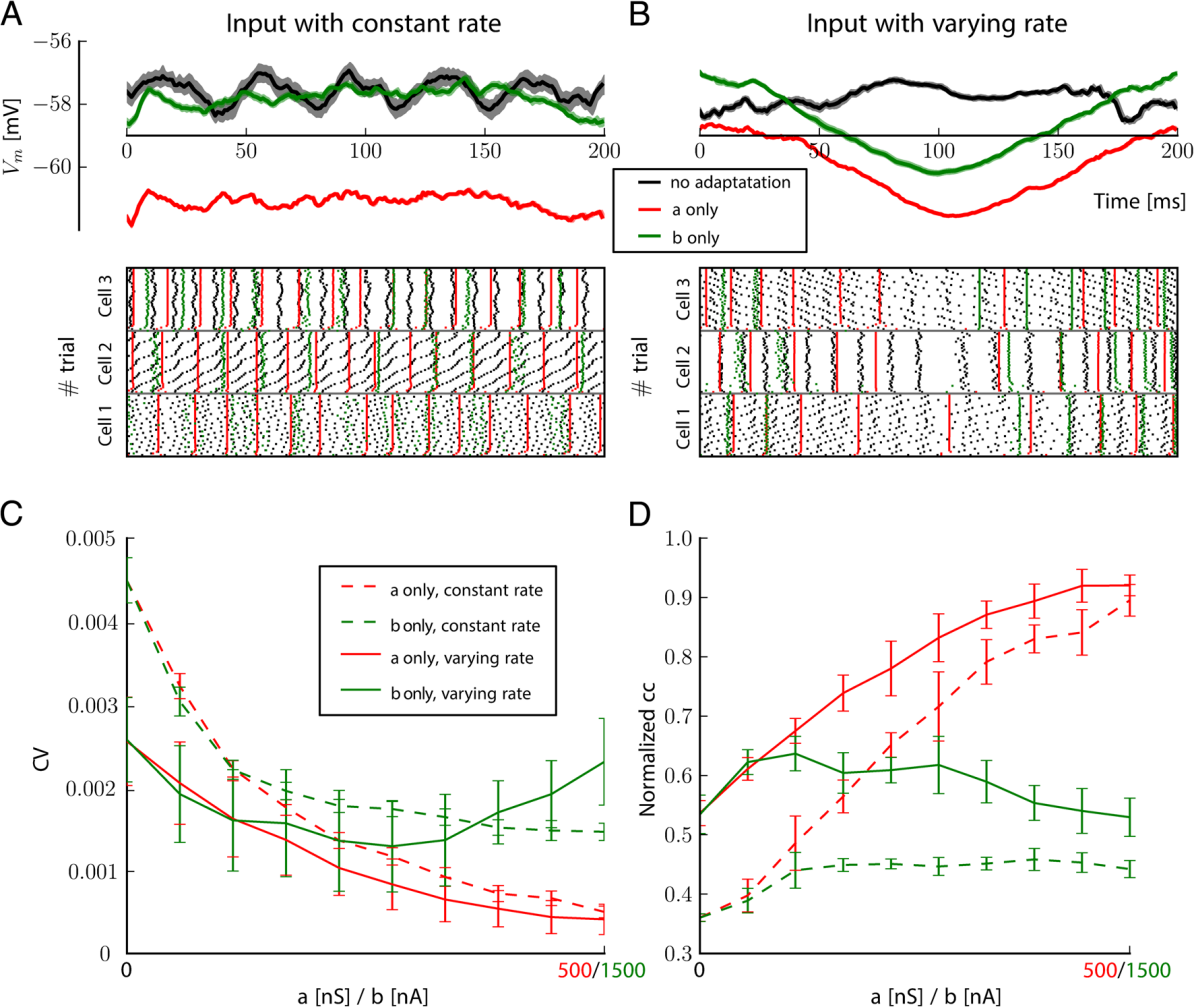
Reliability of columnar dynamics under repeated stimulus. We consider neurons with no adaptation current in black, with only linear (*a* = 500*nS*) in red and with only nonlinear adaptation current (*b* = 1500*nA*). **a** Mean membrane potential ± std averaged over repetitions to a constant Poisson input at 850Hz, without any adaptation (*black*), with linear only (*red*), or non-linear only (*green*). Below are the trial-to-trial raster plots of three representative cells in the column each spiking over 40 repetitions, in those three conditions. **b.** Same as in **a.** but in response to an Poisson process with rate varying as a sinus during a period of 200 ms (see Methods). **c.** Measures of the Coefficient of Variation (CV) of the mean membrane potential in the period for the 40 repetitions of the stimulus averaged across 10 realizations of the network. The measures was computed for constant (*solid*) or varying (*dashed*) input rate as a (*red*) or b (*green*) were varied. **d.** Normalized pairwise cross-correlations between repeated response of a neuron, averaged across 100 non-silent cells and over 10 realizations. *Line* and *color* codes are same as in **c**

On Fig. 9a, b, we can see the temporal dynamics of the mean membrane potential, averaged over 40 repetitions, and when stimulated with constant Poisson input (Fig. 9a) or time-varying inhomogeneous Poisson input (Fig. 9b) (see Methods for a description of the input stimulus). In both constant and varying rate cases, the variance over trials of the responses are reduced by the adaptation currents. This can be viewed more clearly in the raster plots below Fig. 9a and b, showing the trial-to-trial spiking responses of 3 sampled neurons taken in the excitatory population of the column for the 40 repetitions of the same input.

To quantify this increase in reliability between trials, we used two measures. The first one is the CV of the mean voltage averaged over all *N*_*e*_ neurons within the column. We can see on Fig. 9c that this measure is affected similarly by both adaptation currents in the case of the stimulation at constant rate (dashed lines), while it has a minimum at a finite value of nonlinear adaptation current *b* in the case of a temporally varying stimulation (solid green line). For high values of *a*, the CV is almost 0, meaning that the reliability is very high. To rule out the fact that this will depend on the average activity, we also computed the normalized averaged cross-correlation coefficient within trials and among neurons spike trains as described in Methods (see Fig. 9d). On Fig. 9d, we can see similar trends as for the average membrane potential: the normalized correlation coefficient is close to 1 for high values of linear adaptation (red curves) and there is an optimal value of *b* at which maximal reliability is achieved with time-varying input rate.

## Discussion

In this paper, we analyzed the effects of adaptation in the adaptive exponential integrate-and-fire neuron model, distinguishing linear and non-linear mechanisms, related biologically to voltage-gated and calcium-gated channels. Using mathematical observations and comparisons with simulations, we showed how those two distinct components could lead to different changes of the firing rate response curve for this particular neuron model. In single-cell simulations, we found that linear adaptation affects mostly the threshold at which a neuron starts to fire, while non-linear adaptation tends to lower the slope of the response curve. Extending those observations to cortical networks, we studied the role of adaptation onto the dynamics of a cortical column (a so-called random balanced network) with an activity similar to what can be observed *in vivo*. We found that linear adaptation introduces a switch from cortical oscillations to a fixed point of stationary low firing rate while non-linear adaptation preserves cortical oscillations but shifts their frequency to lower values. These are thus two possible ways to modulate synchrony in a neuronal network.

From a functional point of view, we showed that the dynamic range of the response of a cortical column is increased when non linear adaptation currents are included and that there is a shift of the threshold and response to lower values when linear adaptation current is included. These aspects may be important to understand the contrast invariant computations at the neuronal level and predictive aspects of the neuronal response (Deneve 2008; Boerlin et al. 2013). We also demonstrate that when oscillatory dynamics arise in a cortical column, both adaptation currents attenuate these oscillations but in different manners. Our study thus sheds lights on the possible ways to modulate oscillatory dynamics with slow currents which is of crucial interest for cognitive neuroscience (Buzsáki 2010). Moreover, we found that adaptation was also able to greatly increase the reliability of the neuronal responses and as observed *in vitro* (Mainen and Sejnowski 1995) and *in vivo* (Haider et al. 2010), responses are reliable on a trial-to-trial basis. The linear part of the adaptation mechanism used in the integrate-and-fire model considered here was the one mostly involved in this process. Because this linear adaptation is related to voltage-gated channels, we can establish a link between the temporal precision of the responses and the distribution of those channels in neurons. Interestingly, it is known that the repartition of such channels can also be activity-dependent (Lu et al. 2004), so neurons may have mechanisms to adapt their precision as a function of their inputs.

Adaptation is an ubiquitous phenomena in the brain that can spawn multiple time scales: from time constants of several minutes or even hours, it has been shown to be crucial for homeostasis and stability in neuronal network. In this work, we investigated only adaptation mechanisms in the order of hundreds of milliseconds, so relatively close to the membrane time constant. This form of adaptation is known to rely on the kinetics of the voltage or calcium gated channels, and for a more in depth knowledge, see Marder (2012). Therefore, our study focused only on dynamical responses and equilibrium that could be reached within those time scales. Results are observed for random balanced networks with sparse connectivity (Vogels et al. 2005), and it is likely that differences may be found in denser networks, or when the balance between excitatory and inhibitory conductances is reduced (Vreeswijk et al. 1996; Ostojic 2014). All together, unravelling the effect of adaptation in spiking networks is crucial to understand the computation that can be performed by such dynamical systems. Functionally, one could hypothesize that linear (sub-threshold) adaptation, responsible for a shift in the threshold of the response curve is useful to implement contrast invariant responses, while the change in the slope of the response curve induced by non-linear (supra-threshold) adaptation increases the dynamic range of the neuron and softly modulates the oscillatory dynamics. This findings remains to be tested *in vivo*.
